# Differential Genotyping of *Mycobacterium avium* Complex and Its Implications in Clinical and Environmental Epidemiology

**DOI:** 10.3390/microorganisms8010098

**Published:** 2020-01-10

**Authors:** Jeong-Ih Shin, Sung Jae Shin, Min-Kyoung Shin

**Affiliations:** 1 Department of Microbiology, Institute of Health Sciences, College of Medicine, Gyeongsang National University, Jinju 52727, Korea; jung20787@gmail.com; 2 Department of Microbiology and Institute for Immunology and Immunological Diseases, Brain Korea 21 PLUS Project for Medical Science, Yonsei University College of Medicine, Seoul 03722, Korea

**Keywords:** *Mycobacterium avium* complex (MAC), genotyping, pulsed-field gel electrophoresis (PFGE), variable number of tandem repeats (VNTR), mycobacterial interspersed repetitive-unit-variable number of tandem repeats (MIRU-VNTR), repetitive element sequence-based PCR (rep-PCR), clinical epidemiology, environmental epidemiology

## Abstract

In recent decades, the incidence and prevalence of nontuberculous mycobacteria (NTM) have greatly increased, becoming a major worldwide public health problem. Among numerous NTM species, the *Mycobacterium avium* complex (MAC) is the most predominant species, causing disease in humans. MAC is recognized as a ubiquitous microorganism, with contaminated water and soil being established sources of infection. However, the reason for the recent increase in MAC-associated disease has not yet been fully elucidated. Furthermore, human MAC infections are associated with a variety of infection sources. To improve the determination of infection sources and epidemiology of MAC, feasible and reliable genotyping methods are required to allow for the characterization of the epidemiology and biology of MAC. In this review, we discuss genotyping methods, such as pulsed-field gel electrophoresis, a variable number of tandem repeats, mycobacterial interspersed repetitive-unit-variable number of tandem repeats, and repetitive element sequence-based PCR that have been applied to elucidate the association between the MAC genotypes and epidemiological dominance, clinical phenotypes, evolutionary process, and control measures of infection. Characterizing the association between infection sources and the epidemiology of MAC will allow for the development of novel preventive strategies for the effective control of MAC infection.

## 1. Introduction

*Mycobacterium avium* complex (MAC), a slow-growing mycobacterium that inhabits a wide range of sources, such as soil, water, domestic and wild animals, and foodstuffs, causes various forms of disease in humans, other mammals, and birds [1]. MAC can survive and multiply in a wide range of environmental conditions, including low pH, extreme temperatures, chlorine or ozone treatment, and low oxygen levels, and thus thrive in various environments due to their ability to utilize various substances as nutrients [2,3]. Moreover, MAC is known to cause a variety of diseases, including tuberculosis-like diseases in humans and birds, disseminated infections in immunocompromised patients, lymphadenitis in humans and mammals, and chronic enteric disease in ruminants [2,4]. In addition, the *M. avium* subspecies, *paratuberculosis* (MAP), a causative agent of Johne’s disease in ruminants, has been identified in specimens from Crohn’s disease, and has been proposed to be related to each other [5]. The prevalence of nontuberculous mycobacteria (NTM) lung disease has recently increased across the world, wherein MAC has been the predominant mycobacterium in most countries, responsible for 47% of the cases of NTM infection [6,7]. In addition, a study published in the early 1990s revealed that the incidence of MAC in AIDS patients ranged from 20–40% [8,9]. These findings indicate that MAC is a representative microorganism among NTM species which cause human disease.

Since public health authorities do not typically monitor NTM disease, relevant epidemiological and surveillance data are not readily available or are inaccurate, indicating that defining the epidemiology of NTM is more difficult than *Mycobacterium tuberculosis* (MTB) [7]. Unlike MTB, there is no definite data indicating the transmission of bacteria between humans, such that the concept that it is acquired from the environment takes precedence in NTM [7]. Environmental infectious agents may have an impact on the specific infection and transmission pathways of MAC, potential infections, and disease recurrence; however, these have not yet been fully defined. Although various epidemiological studies on MAC have been conducted using different genotyping techniques, there is still a lack of literature presenting the epidemiological characteristics, biology, and origins of MAC from humans, animals, and environmental sources using definitive genotyping techniques. As such, in this review, we discuss the application of the major genotyping methods for the characterization of MAC, including restriction fragment length polymorphisms (RFLP), pulsed-field gel electrophoresis (PFGE), variable number of tandem repeats (VNTR), mycobacterial interspersed repetitive-unit (MIRU)-VNTR, and repetitive element sequence-based PCR (rep-PCR), and deal with the associations of genotypes with epidemiological investigation, diagnosis, clinical phenotypes, evolution, transmission mode, and prevention of MAC in order to control these organisms more effectively.

## 2. Significance of Molecular Genotyping Methods Applied to MAC

Traditionally, MAC is thought to consist of two species, *M. avium* and *M. intracellulare* [10,11]. Recent advances in systematic analysis have allowed for the identification and classification of new (sub) species within MAC at the molecular level, such as *Mycobacterium chimaera* [12], *Mycobacterium colombiense* [13], *Mycobacterium arosiense* [14], *Mycobacterium vulneris* [15], *Mycobacterium bouchedurhonense*, *Mycobacterium marseillense*, *Mycobacterium timonense* [16], and *Mycobacterium paraintracellulare* [17], as well as newly defined *M. intracellulare* subspecies, including *M. intracellulare* subsp. *yongonense* [18,19] and *Mycobacterium indicus pranii* [19]. Additionally, *M. avium* has been divided into four subspecies: *M. avium* subsp. *avium* (MAA) [20], *M. avium* subsp. *hominissuis* (MAH) [21], *M. avium* subsp. *paratuberculosis* (MAP), and *M. avium* subsp. *silvaticum* (MAS) [20]. With the development of molecular diagnostic methods, novel species have been recently identified and published within the MAC family, as phenotypic tests do not readily distinguish between closely-related species and subspecies [22]. An important example of identifiable phenotypic tests in MAC is the fact that MAP requires mycobactin J for in vitro growth, and MAS is unable to grow in an egg-based medium, being stimulated to grow by pyruvate instead [23]. Due to the difficulty involved in performing species identification using these phenotypic methods, attempts have been made to characterize MAC classification using molecular genotyping methods, such as sequence analysis of specific targets, detection of species-specific insertion elements, and restriction enzyme analysis, thus providing new methods for the identification of novel MAC species and strains among others, providing new opportunities for the identification of novel MAC species and strains [23].

MAC differs in virulence and ecology within the constituent bacteria, among which species such as MAA, MAP, MAH, and MAS are strict pathogens; however, *M. intracellulare* is considered to be an environmental bacterium that is widely distributed in soil and water [24]. Additionally, a new related species and a new subspecies of *M. intracellulare* were isolated and identified in patients with pulmonary disease, lymphadenitis, or disseminated infection; however, isolation from the environment or animals was not reported. Later, the mechanical meaning of MAC will be described in detail, but in short, *M. avium* can be excreted from infected animals and contaminate the environment, so that it is considered as possible to be transmissive among humans, animals, and the environment. However, *M. intracellulare* has been described as being in the environment, such as soil and water, and its infection has been attributed to the environment. Thus, there have been discussions that the two bacteria must not be bound together by MAC because of different infectious sources [1,25]. Among other factors, water is considered to be the main source of MAC infection in humans [26]. Indeed, MAC has also been detected in samples of hospital water distribution systems [27]. It is believed that drinking water systems contribute to the dissemination of MAC infection [28,29]. A statistically significant correlation between freshwater exposure of patients and MAC infection has been reported; however, the studies did not report a microbiological association, and only partial evidence for direct transmission was observed [30,31]. Thus, while MAC is an important public health pathogen, since it resides in the environment, animals, and humans, the epidemiology of MAC organisms remains ambiguous.

To date, the optimal approach by which to determine the epidemiology of MAC and characterize the risk factors associated with sources of infection is molecular genotyping. Although the sequencing of 16S rRNA genes is key to the identification of MAC at the species level, the sequencing of other target genes, including *rpoB*, *hsp65*, and the internal transcribed spacer (ITS) has not yet been determined for the genotypes of all MAC organisms [22]. Therefore, RFLP, PFGE, and VNTR analysis have been used to distinguish MAC isolates, wherein various PCR-based analysis that can be highly reproducible and much faster than these methods have been developed and used. New strains are constantly being classified as MAC species or subspecies, and their infectious sources may be more varied than the initial expectations. In particular, the data on the MAC genotyping analysis of the MAC obtained thus far is extensive, but not clear due to the genetic characteristics of the species/subspecies, which have not yet been clearly defined. Therefore, it is necessary to prepare criteria and a basis for the selection of the appropriate molecular genotyping methods for each MAC organism by summarizing the discrimination power and epidemiological results derived from each molecular genotyping method for MAC isolates. In the following sections, we discuss several genotyping methods, including PFGE, VNTR, VNTR-MIRU, and rep-PCR, which are representative and widely applied to mycobacterial species, including MAC, and describe the epidemiological results obtained so far.

## 3. PFGE for MAC and Their Implications in Epidemiological Studies

PFGE recognizes and cuts specific bacterial genomic DNA sequences using restriction endonuclease, and effectively separates large DNA fragments by periodically changing the direction of the electric field [32]. The unique band patterns in PFGE are generated either by mutations that create or remove enzyme breakpoints, or by genetic changes, like deletions or insertions, which increase or decrease the band sizes [33]. Although PFGE is labor-intensive and requires a high level of operator skill, it is considered the “gold standard” of genotyping in epidemiological studies of bacterial infections, including MAC [34,35,36]. The usefulness of PFGE analysis in epidemiological studies is described below.

### 3.1. Investigation of MAC for Co-Infection and Relapse

Arbeit et al. (1993) reported on a case in which a patient was simultaneously infected with two different strains. Additionally, the study addressed the need for alternatives for the diagnosis and treatment of co-infection cases [34]. The analysis of the isolates from patients with AIDS revealed that 33% of the patients had polyclonal infections. The cases tested positive for both *M. avium* and *M. intracellulare*, which indicated that co-infection is common among patients with AIDS [37]. Additionally, the analysis of MAC infection profiles based on the symptoms revealed that polyclonal MAC infection was common in patients with nodular bronchiectasis, whereas monoclonal infection was more common in patients with fibrocavitary lung disease. Interestingly, *M. intracellulare* infection was dominant in both nodular bronchiectasis and fibrocavitary lung disease [38].

Among the co-infection cases, the study of recurring MAC pulmonary disease began with the analysis of *M. avium* infection in patients with AIDS [39]. Three strains were sequentially isolated from one patient, wherein each strain exhibited varying resistance to clarithromycin, despite all three strains being found in the same patient. Additionally, 97.4% of the patients who exhibited a positive culture after 2–15 months of treatment had the same strains as those before treatment [39]. This study suggests that *M. avium* relapse in patients with AIDS may be caused by the same strain acquiring resistance to antibiotics or due to the reappearance of latent strains present in the tissue [39]. Wallace et al. (2002) conducted a follow-up study of MAC recurrence after macrolide treatment, in which only 4 out of 36 cases of MAC were infected by the same strain [40]. Additionally, 5 of the 7 MAC relapse cases observed after ending the treatment too early were caused by the same strains, wherein the newly infected strains were not resistant to macrolide treatment [40]. These findings indicated that the macrolide treatments in these patients were complete and that the relapse from the same strain was rare, most likely being caused by different strains [40]. This was similar to the findings of Jhun et al. (2018) using rep-PCR, demonstrating that the continuous influx of bacteria and infection is the main issue, rather than the recurrence of the same bacteria [41].

### 3.2. Relationship among Environment, Animal, and Human Isolates

A study was conducted on the assumption that the residence of the patients studied was a contributing factor to the relapse of MAC infection, as MAC was found only in the bathroom within their residences [31]. Additionally, the detection rate of MAC in the patients’ bathrooms was significantly higher than that in the general public space. Of the 49 patients, two patients had the same strains as those detected in their bathroom [31]. Several other studies have also examined the correlation between strains of MAC detected in living quarters and the isolates obtained from the patients [42,43]. In particular, the strains detected in one patient with hypersensitivity pneumonitis who was occupationally exposed to water used for cleaning the pool filter matched the strains detected in the workplace [44]. Additionally, studies on MAC infection caused by aerosols generated by heater-cooler devices used in research and heart surgery revealed that devices used in medical and research facilities can be sources of MAC infection, indicating the possibility that these types of MAC infection will continue in the future, despite the development of hygiene or modern medicine [45].

The study of the infectious environment of *M. avium* compared the genotypes of strains found in reservoirs, households, commercial buildings, hospitals, and clinical isolates in Los Angeles and California. The highest correlation was observed between the isolates obtained from hospitals and patients, indicating in-hospital infection. This supported the hypothesis that *M. avium* infection is possible through drinking water [46]. Subsequently, Kyriakopoulos et al. (2000) reported that infection in patients was caused by exposure to an environment containing the related strains rather than by clinical features, as the strains of similar genotypes were isolated in both HIV-negative and -positive patients. This study addressed the need for further analysis [47].

Drovska et al. (2003) used MAA and MAS isolation panels from 27 different hosts and environments to identify MAA (RFLP type F-C3) in all birds diagnosed with avian tuberculosis [48]. MAA (RFLP type F-C3) was also found in the aviary environment [48]. This study showed that the MAA of a specific RFLP type could be introduced in a flock and subsequently spread rapidly among susceptible birds, resulting in the establishment of a potentially infectious environmental reservoir [49]. In another study, MAA was isolated from captive water birds and from environmental samples from aviaries housing naturally infected captive water birds. The results confirmed that these strains were equally virulent in pullets [49]. These results suggested that the local environment could spread MAA—the causative agent of avian tuberculosis—among zoo and farm animals, as well as their caregivers [49]. As discussed above, several studies have confirmed that MAA excreted from MAA-infected animals (i.e., in feces) contaminate the environment and that the environment might play a role in MAA transmission.

Meanwhile, interestingly, when by-products of hens were fed to second-stage larvae of the blowflies *Calliphora vicina* and *Lucilia sericata*, MAA was recovered from the *C. vicina* and *L. sericata* larvae four days after infection [50]. Moreover, in the same study, MAP (RFLP type B-C1) were detected in two samples including the intestinal mucosa of two cows showing clinical signs of Johne’s disease, and captured blowfly *C. vicina* that was in contact with the intestinal mucosa present in the slaughterhouse waste container [50]. In addition, MAP was also detected in captured blowflies on the following day, in the absence of cows with Johne’s disease [50]. These results showed that both the vector (the blowfly) and the larvae of the vector may participate in spreading the agents of mycobacterial infection, reinforcing the importance of proper hygiene during handling animals infected with, or affected by, MAA or MAP [50].

Feizabadi et al. (1996) evaluated the association between human and animal isolates using multilocus enzyme electrophoresis (MEE). A low correlation between bird and human isolates, and a high correlation between pig and human isolates were reported [51]. The results of MEE and PFGE showed that certain strains of *M. avium* could be transmitted between birds and pigs. However, there was no definite evidence of transmission to humans [51]. Johansen et al. (2007) reported that MAH pig isolates showed regional differences, but no differences in terms of clinical type or region in genotyping analysis on MAH human and MAH pig isolates [52]. There was a significant difference in genotype between the MAH human and MAH pig isolates, although one human isolate was identical to the pig isolate [52]. IS*1311* analysis and PFGE analysis using 520 MAPs isolated from several animals showed that the bovine isolates had a similar genotype, while those from sheep and goats were different [53]. Based on the above results, genotypes could be classified according to host specificity within the same species.

However, Tirkkonen et al. (2010) demonstrated significant similarities between MAH human and MAH pig isolates [54]. Hence, further investigations need to be done to determine whether one species (human or pig) can alternately become a source of infection for another species, and whether the infection has been transmitted through common environmental sources. These results might offer insight into the epidemiology of MAH among humans and pigs. Major sources of porcine MAH infection are believed to be peat and sawdust, which are frequently used as bedding in the swine industry [55,56,57,58]. However, recent results indicated that the role of feces and peat in swine infections cannot be defined accurately. Nonetheless, the detection of MAH in feces of naturally infected and experimental pigs offers support for MAH infection by the fecal–oral route [59,60]. Moreover, human MAH infection was traced to drinking water systems, saunas, pools, and organic environmental substances [28,46,60,61], which can cause severe disseminated infections in immunocompromised patients, such as those infected with HIV [3,10].

### 3.3. Comparison of PFGE with Other Genotyping Methods

PFGE, IS*1245*-PCR, and IS*1311*-PCR analyses revealed that *M. avium* isolates from a hospital exhibited varied genotypes. The IS element-PCR analysis detected 2–3 patterns, whereas PFGE analysis detected 5–6 groups, indicating the limitations of IS element analysis [62]. *M. intracellulare* isolated 21 from patients suffering from bronchiectasis were analyzed by VNTR and PFGE analysis. The patterns 22 of the two analyses were similar, suggesting that VNTR may be a suitable alternative to PFGE for use in distinguishing recurrence [63]. In addition, *M. intracellulare* was subjected to antibiotic susceptibility testing, sequencing of the *hsp65* and *rpoB* genes, PFGE, Multilocus sequence typing (MLST), MIRU-VNTR, and VNTR [64]. The discrimination power for *M. intracellulare* was found to be higher in order of PFGE, VNTR, MLST. Furthermore, it was reported to be useful for distinguishing recurrence [64].

In more recent studies, PFGE and whole-gene sequencing (WGS) have been used in combination. After the sequential isolation of bacteria from chronic patients, one strain was continuously infected with PFGE. Interestingly, WGS analyses using selected strains showed that one strain adapted to the host due to chronic infection, wherein the down-regulation of inflammatory cytokines in the host associated with mycobacterial infection occurred as mutations accumulated [65].

## 4. VNTR for MAC and Their Implications in Epidemiological Studies

Variable number tandem repeat (VNTR) is a genotyping method that analyzes the band size after PCR amplification and electrophoresis using primers specific for the region surrounding the VNTR sequence [66]. Since the repeat unit length of tandem repeats is known, this band size allows for the calculation of a number of different VNTR copies per strain, ultimately presenting the data as the number of VNTR repeats at each locus [67]. These quantitative data are particularly useful for comparative studies within and between laboratories and countries. This includes the VNTR of a genetic element called mycobacteria-interspersed repetitive units (MIRUs), which are scattered throughout the genome of MTB, although they are predominantly located in the internal regions [66,67]. In molecular epidemiology studies of *M. avium*, VNTR genotyping provides an alternative to IS*1245* RFLP genotyping [68]. This is because VNTR has higher reproducibility compared to the short-term changes of IS*1245* elements and does not require high DNA purity, as in RFLP genotyping. Moreover, the discriminatory index of VNTR genotyping is similar to or higher than that of IS*1245* RFLP genotyping [68]. However, previous studies have suggested the use of RFLP genotyping in combination with VNTR genotyping, as there are strains that could be identified by IS*1245* RFLP genotyping but not VNTR genotyping [68]. The following subsections detail the identification of VNTR and MIRU loci, the development of genotyping techniques using these loci, and the epidemiological indications.

### 4.1. Identification of VNTR Loci and Development of VNTR Techniques

In 2007, the MIRU-VNTR locus was identified using the genome sequence of the MAP K10 strain for the study of MAP isolates [69]. Strain identification was validated not only in MAP, but also in *M. avium* by IS*1245* RFLP genotyping [39]. Comparative MIRU-VNTR analysis of *M. avium* and MAP revealed that the 183 MAP isolates were grouped into only 21 types, whereas 82 *M. avium* isolates were grouped into 30 types without overlapping patterns with MAP, demonstrating that the newly developed MIRU-VNTR genotyping technique had a high discrimination power for *M. avium* [69]. However, as this genotyping method was developed using the MAP strain, there was a need to find another locus to distinguish the MAC isolates, which led to the development of a new MATR-VNTR primer. MATR-VNTR was generated based on several VNTR markers with the genetic information of MAH 104 and MAP K10 [69,70,71,72]. MATR-VNTR genotyping resulted in a higher detection of *M. avium* than MIRU-VNTR genotyping [68].

The VNTR locus was also selected using the *M. intracellulare* ATCC 13,950 standard strain, which was applied to the *M. intracellulare* clinical isolates to confirm the high degree of identification [73]. Additionally, MIRU markers for *M. intracellulare* were established. Polymorphisms in the *M. intracellulare* isolates and standard strain were identified using MIRU 1 and 4, which were established by [70] using *M. avium*, MIRU 32, 292, X3, 25, 3, 7, 10, and 47 using MAP by [69], and all 16 loci from MAV 104, and 17 loci from *M. intracellulare* ATCC 13,950 by [74]. The following seven loci, except for those that were not amplified or mutated, were identified and confirmed to be stable in 10 passages in medium: MIRU 3 [70], MIN 18, MIN 19, MIN 20, MIN 22, MIN 31, and MIN 33 (derived from *M. intracellulare* ATCC 13950) [74]. The detailed information of the studies involved genotyping and discriminant analyses of MAC using VNTR and MIRU are summarized in Table 1. 

The maximum Hunter–Gaston discriminatory index (HGDI) value was 0.172 using four MIRUs, three TRs, and one MATR loci for MAS, 0.567 using four MIRU, and three TRs loci to 0.751 using eight TR loci for MAP. *M. avium, M. intacellulare*, MAA, and MAH displayed an HGDI of 0.723 to 0.999 in combination with various MIRU, MATR, and TR loci, indicating that VNTR genotyping of *M. avium, M. intacellulare*, MAA, and MAH showed higher HGDI compared to MAS and MAP (Table 1). In particular, in several studies using VNTR genotyping analysis applied to *M. avium*, MAA, MAH, and MAP, allelic diversity for each MIRU, TR, RD, and MATR loci was obtained. The data are summarized in Table 2. When allelic diversity (*h*) of VNTR loci was presented in the previous studies, the information was presented directly in Table 2. However, if the *h* value was not presented directly in the literature, we extracted the VNTR type results from the results or supplementary data of the studies and calculated their *h* values using these results. Genetic diversity at the locus extracted from each reference was calculated as follows:

Allelic diversity index (*h*) = (1 − ∑xi^2^) [*n*/(*n* − 1)], (0 ≤ *h* ≤ 1) (1)

where Xi is the relative frequency of the i-th gene locus, *n* is the total number of samples, and *n*/(*n* − 1) is a value to correct the error due to the small number of samples [75].

As shown in Table 1, the HGDI of the VNTR technique was different depending on the species, especially the combination of loci. Therefore, we present the allelic diversity (h) for each locus in Table 2, and compared the allelic diversity for each locus according to infection sources, geographic regions, and species. Few studies have used the MIRU locus compared to other loci, but showed different *h* values for each species. In particular, MAP showed that each locus had a generally low *h* value, so that the MAP genotype was less diverse than other species, or that the developed VNTR technique had low discrimination power for MAP. Moreover, Iwamoto et al. (2012) analyzed MAH isolates from humans, the environment, and animals using MATR loci. The *h* of each locus showed similar trends in human and environmental isolates, and animals showed different *h* value trends. However, animal isolates obtained in Switzerland tended to have different *h* values for each locus compared to the animal isolates from Japan. Therefore, the genetic diversity of loci depending on the source of infection is considered irrelevant. This information will be helpful for the selection of locus suitable for research purposes when applying VNTR and VNTR-MIRU genotyping techniques to MAC.

### 4.2. Application of VNTR Method for Clinical and Epidemiological Investigations

MIRU-VNTR analysis was performed using various sources of MAP 316F strains used for vaccination. The identification of the strains used in vaccines is necessary, as there are large variabilities within the same 316F strains [69]. In 2009, the association between the clinical features and molecular epidemiology of the different strains was studied using the previously developed MATR-VNTR genotyping technique [76]. In this study, the progression of lung disease caused by *M. avium* was associated with a specific VNTR cluster. Additionally, among the three VNTR groups of clinical isolates, isolates from patients with progressive disease were more likely to be in group C, whereas those from patients with stable disease were more likely to be found in group A. Although the VNTR genotype cannot determine the presence of lung disease, it can predict the progression of lung disease caused by *M. avium* infection [76].

The study used a combination of MIRU and VNTR loci, and the association of VNTR clusters, which had clinical significance. NTM infection and the presence of cavity were also related to the VNTR type [77]. Additionally, the study confirmed the similarity between VNTR and PFGE profiling of *M. intracellulare* patient isolates using seven MIRU loci. The combination of VNTR and 16S multiplex PCR allowed for the determination of disease relapse [63]. The MATR loci were used to study the isolates obtained from patients with pMAH135 plasmids containing pathogenic and antibiotic resistance genes in MAH, which influence host specificity [78]. Additionally, the ability to be infected by human macrophages and to proliferate were reported to depend on the VNTR type of MAH using MIRU loci [79].

Moreover, MATR-VNTR genotyping was also used to evaluate the transmission between patients with *M. avium* and the surrounding environment in which *M. avium* exists [80]. In this study, both *M. avium* isolated from soil obtained from the patient’s residence and *M. avium* from the patient were subjected to MATR-VNTR genotyping. The analysis revealed that there was a higher possibility of two VNTR patterns matching if the patient was exposed to the soil [80]. This suggested that *M. avium* may be transmitted directly to patients via the soil from its habitat to the patient’s residence [80]. Additionally, a follow-up study also confirmed that the frequency of exposure to environmental sources of *M. avium* was directly proportional to the number of detected *M. avium* isolates with various VNTR patterns (polyclonal). This indicated that *M. avium* can be transmitted to patients via environmental exposure [81]. The correlation between contact with the infected environmental and MAC infection was analyzed even in the case of *M. intracellulare* [80,81]. 

**Figure 1:**
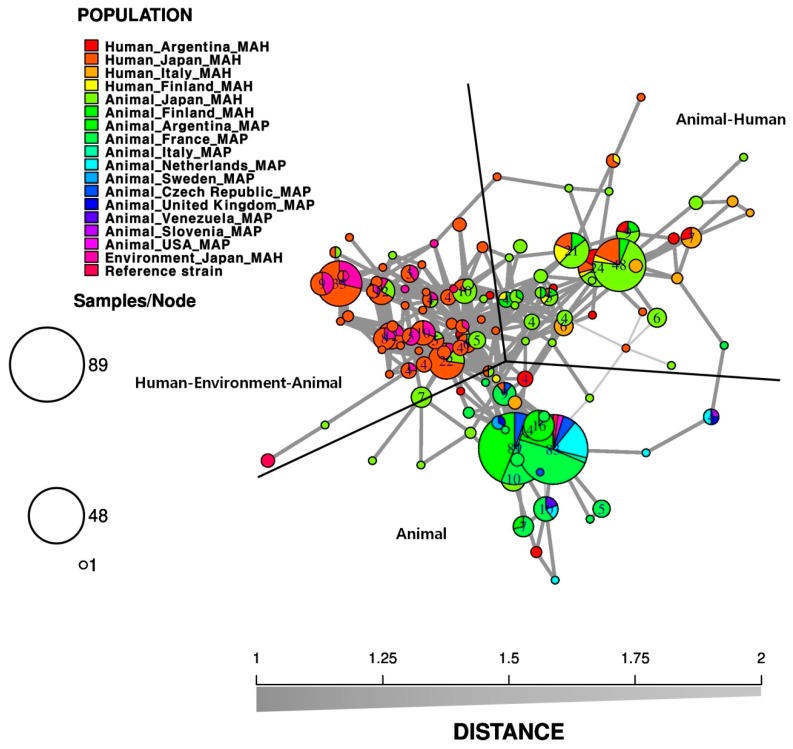
A minimum spanning tree (MST) based on 7−TR (TR 292, X3, 25, 47, 7, 10, and 32) genotyping for *M. avium subsp. hominissuis* (MAH) and *M. avium subsp. paratuberculosis* (MAP) isolates from different geographic regions and sources. The strains used in this analysis were as follows: isolates from Human−Argentina−MAH (*n* = 22, [95]), Human−Japan−MAH (*n* = 169, [96]), Human−Italy−MAH (*n* = 22, [79]), Human−Finland−MAH (*n* = 13, [54]), Animal−Japan−MAH (*n* = 141, [96]; *n* = 12, [88]), Animal−Finland−MAH (*n =* 16, [54]), Animal−Argentina−MAP (*n* = 14, [69]; *n* = 61, [95]), Animal−France−MAP (*n* = 116, [69]), Animal−Italy−MAP (*n* = 2, [69]), Animal−Netherlands−MAP (*n* = 27, [69]), Animal−Sweden−MAP (*n* = 2, [69]), Animal−Czech Republic−MAP (*n* = 12, [69]), Animal−UK−MAP (*n =* 2, [69]), Animal−Venezuela−MAP (*n =* 2, [69]), Animal−Slovenia−MAP (*n =* 1, [69]), Animal−USA−MAP (*n =* 2, [69]), Environment−Japan−MAH (*n =* 37, [96]), and reference strains (MAP K10 and MAP ATCC 19698, [69]; MAA ATCC 15769, MAA ATCC 25291, and MAA ATCC 35712, [54]). Each circle corresponds to the VNTR genotype, and the size of the circle is proportional to the number of strains showing the same pattern. We performed MST analysis based on VNTR genotypes using the poppr package of the R software (version 2.1.0) [82] to reconstruct a hypothetical phylogenetic tree for the MAH and MAP isolates.

In this study, minimum spanning tree (MST) analysis was performed by extracting the results obtained using TR loci from the references cited in Table 1 and Table 2. Using the extracted TR loci copy numbers, MST results were obtained using the poppr package of the R software (version 2.8.3,) [82]. MST analysis was performed using MAH and MAP isolates according to infection sources and geographic regions. As shown in Figure 1, MAP had only animal isolates, but there was no genetic difference according to the geographic regions. In addition, MAH human and animal isolates were either displayed the same genotype, or the isolates were located close to the cluster where the MAP isolate was located. The results proved that MAH and MAP are genetically close. A comparative genomic analysis of MAH reported that MAP already showed a reduced genome size and decreased levels of genetic variability [83]. Thus, MAP is believed to be a host-adapted pathogen, which evolved from MAH via genetic loss and acquisition [83,84]. MAH isolates were also divided into two large clusters, one clustered with human, environment, and animal isolates, and the other with animal and human isolates. Therefore, genotyping using TR loci could confirm the epidemiological relationships between humans, the environment, and animals (Figure 1).

### 4.3. Geographical Relationship between Bacterial Strains Belonging to MAC

VNTR genotyping can also be used to study the association between strains. In particular, VNTR genotyping is more suitable for the identification of MAA, MAH, and MAS than IS*1311* RFLP genotyping [85]. VNTR genotyping supports the hypothesis that MAA, MAP, and MAS evolved independently in MAH [85]. However, for MAS, the variation between strains could not be confirmed by VNTR genotyping [85]. Subsequent epidemiological studies using MATR markers confirmed the association of MAH in isolates obtained from Dutch, German, US, Korean, and Japanese patients, and found that the Japanese strains were genetically closer to the Korean strains than the European or American strains [86]. Interestingly, the geographic origins or genetic associations of *M. intracellulare* were not significantly related [86].

The association between animal and human isolates was also investigated. In the VNTR genotyping analysis using MATR and MIRU loci, some MAH Swiss isolates from bovine lymph nodes were similar to the pulmonary patient isolates from Netherlands, United States, and Japan [87]. The IS*Mav6* gene, which is distributed predominantly among isolates obtained from a patient in East Asian regions, was first found in isolates obtained from animals, indicating a close genetic association between the human and animal strains [87]. Additionally, a comparison of VNTR types analyzed using MIRU and MATR loci in previous studies of MANT BVLA01 strains isolated from cattle showed a close relationship between isolates obtained from Japanese patients and from the bathtub [88]. That is, it was possible to confirm the relationship between humans, the environment, and animal isolates, and not to have a specific genotype indicating host specificity in MAH.

**Figure 2:**
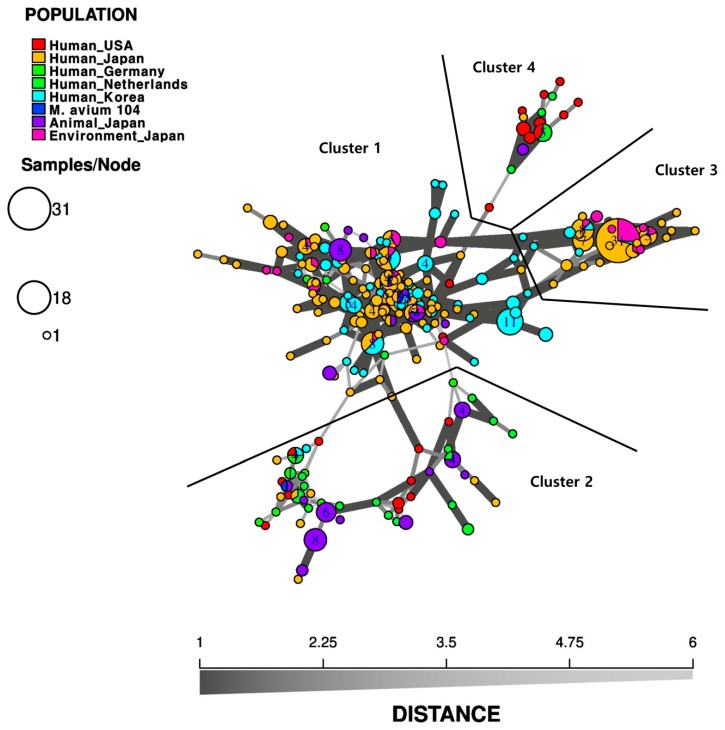
A minimum spanning tree (MST) based on 14−MATR (MATR 1 to 16 excluding 9 and 10) genotyping for *M. avium subsp. hominissuis* (MAH) isolates from different geographic regions and sources. The strains used in this analysis were as follows: isolates from Human−USA (*n =* 32, [86]), Human−Japan (*n =* 142, [96]; *n =* 94, [86]), Human−Germany (*n =* 10, [86]), Human−Netherlands (*n =* 27, [86]), Human−Korea (*n =* 98, [86]), Animal−Japan (*n =* 45, [96]; *n =* 12, [88]), Environment−Japan (*n =* 37, [96]), and M. avium 104. Each circle corresponds to the VNTR genotype, and the size of the circle is proportional to the number of strains showing the same pattern. We performed MST analysis based on VNTR genotypes using the poppr package of the R software (version 2.1.0) [82] to reconstruct a hypothetical phylogenetic tree for the MAH isolates.

In this study, MST analysis was performed by extracting the results derived from 14-MATR loci (MATR 1 to 16 excluding 9 and 10) from the references cited in Table 1 and Table 2. MST analysis was performed using MAH isolates according to the geographic regions. As shown in Figure 2, clusters 2, 3, and 4 branched out around cluster 1, where the Korea-human and Japan-human isolates and some Japan-environmental and Japan-animal isolates were occupied. Cluster 2 consisted of Japan-animal isolates, United States, Germany, Netherlands-human isolates, and Japan-animal isolates. Cluster 3 consisted of Japan-human and Japan-environment isolates, indicating no genetic difference among isolates according to the infection source. In cluster 4, mainly United States and Netherlands-human isolates had clustered, and genotyping analysis using MATR loci showed a rough regional discrimination (Figure 2).

### 4.4. Comparison of VNTR Using Other Genetic Typing Methods

Meanwhile, one study demonstrated that, when using the MIRU marker—which is considered to be less effective than MATR—in combination with CCG-PCR, a type of rep-PCR, the identification ability improved. In addition to the combination of NTR and the IS element RFLP genotyping method previously recommended by Thibault et al. (2007) and Inagaki et al. (2009), a new method that complements the MIRU marker with a new genotyping method was proposed [68,69,89].

Park et al. (2018) classified bison-type MAP strains isolated from Korea, which were identified only by INMV 68 in the MIRU-VNTR typing, into three subtypes by MIRU-VNTR and eight subtypes by multilocus short sequence repeat (MLSSR) typing [90]. The HGDI values in the MIRU-VNTR and MLSSR were calculated to be 0.567 and 0.866, respectively, suggesting the combination of MLSSR typing in MAP genotyping [90]. Additionally, and as described in this manuscript, MAP suggested that each locus had a generally low *h* value, so that MIRU-VNTR typing could not provide a sufficient epidemiological implication for MAP. Although MIRU-VNTR typing does not yield good discernment in MAP and cannot be applied equally to all MACs, the epidemiological meanings can be obtained by using VNTR data for MAC performed globally, as we show presently. 

**Table 1:** List of MIRU-VNTR loci and their discrimination power used for MIRU-VNTR typing applied to *Mycobacterium avium* complex.

Strain	Origin		Sample No.	VNTR Type	Loci No.	HGDI ^1^	Reference
	Source	Country					
***M. avium***	AIDS patients	France	82	30 types	8 TRs	0.889	[69]
	Patients with/without pulmonary disease	Japan	40	27 types	16 MATRs	0.945	[76]
	HIV-negative patients with pulmonary MAC infection	Japan	70	56 MATR, 27 TR types	15 MATRs, 8 TRs	MATR: 0.990 TR: 0.949	[68]
	Patients	Poland	33	21 types	8 TRs	0.945	[89]
	Patients with pulmonary MAC infection and residential soil samples	Japan	88	78 types	15 MATR	0.997	[80]
	Patients with pulmonary MAC infection	Japan	310	93 types	15 MATR	0.987	[81]
	Patients with pulmonary NTM infection	China	41	29 types	13 MATRs	0.993	[91]
**MAA**	Bird, poultry, pig, wild animal, cat, bovine, goat	France	31	8 types	8 TRs	0.723	[85]
	Diseased cattle, slaughtered pigs	Germany	27	19 types	6 MIRUs, 2 VNTRs, 6 TRs, and 1 RD	0.966	[92]
	Wild and domestic mammals, reptiles and birds	Hungary	135	16 types	4 MIRUs, 3 TRs, and 1 MATR	0.845	[93]
**MAH**	Patients (HIV positive and negative), pig, bovine, kangaroo, wild animal, soil sample	France	82	23 types	8 TRs	0.807	[85]
	Diseased cattle, slaughtered pigs	Germany	16	15 types	6 MIRUs, 2 VNTRs, 6 TRs, and 1 RD	0.992	[92]
	Patients	Italy	47	8 types	8 TRs	0.862	[94]
	Patients with pulmonary MAC infection (HIV positive and negative)	Japan	64	55 types	15 MATRs	0.995	[78]
	Wild and domestic mammals, reptiles and birds	Hungary	84	33 types	4 MIRUs, 3 TRs, and 1 MATR	0.966	[93]
	Patients	Argentina	26	16 types	8 TRs	0.93	[95]
	Patients	Italy	23	8 types	8 TRs	0.870	[79]
	Slaughtered cattle	Switzerland	26	14 types	15 MATRs, 5 TRs	0.972	[87]
	Slaughtered bovine with abnormal pulmonary case	Japan	12	9 types	7 TRs, 14 MATRs	0.955	[88]
	Humans, pigs and bathroom environments	Japan	258	150 types	7 TRs, 15 MATRs	0.987	[96]
**MAP**	Bovine, goat, ovine, cervine, and leporine	Argentina, Czech Republic, France, Italy, Netherlands, Slovenia, Sweden, United Kingdom, USA, and Venezuela	183	21 types	8 TRs	0.751	[69]
	Cattle, sheep, goat, wild boar, red deer, red fox, buffalo, mouflon, swine	Denmark, France, Germany, Hungary, Italy, Netherlands, and Slovakia	515	15 types	4 MIRU, 3 TRs	0.598	[97]
	Cattle	Argentina	61	5 types	8 TRs	0.6984	[95]
	Cattle	Korea	27	4 types	8 TRs	0.567	[90]
**MAS**	Wood pigeon	France	4	1 type	8 TRs	0	[85]
	Wild and domestic mammals, reptiles and birds	Hungary	62	5 types	4 MIRUs, 3 TRs, and 1 MATR	0.172	[93]
***M. intracellulare***	Patients	France	62	44 types	7 MIRUs	0.98	[74]
	HIV-negative patients with pulmonary disease	Japan	74	50 types	16 VNTRs	0.988	[73]
	Patients with pulmonary MAC infection and residential soil samples	Japan	55	53 types	16 VNTRs	0.999	[80]
	patients with nodular bronchiectasis	USA	176	42 types	7 MIRUs	0.978	[63]
	Patients with pulmonary MAC infection	Japan	74	27 types	16 VNTRs	0.970	[81]
	Patients with pulmonary MAC infection	Japan, Korea, Netherlands, and USA	116	82 types	16 VNTRs	0.988	[86]
	HIV-negative patients with pulmonary disease	China	77	69 types	1 MIRU, 7 VNTRs	0.997	[77]
	Patients with pulmonary NTM infection	China	132	88 types	16 VNTRs	0.995	[91]

^1^ HGDI, Hunter-Gaston discriminatory index.

**Table 2:** Allelic diversity index of MIRU-VNTR loci for *Mycobacterium avium* complex ^1^.

		*M. avium*					MAA		MAH											MAP			
Sources		Human	Human	Human	Human/Environment	Animal	Animal	Animal	Human/Animal	Human	Human	Animal	Animal	Animal	Human	Human	Environment	Animal	Human/Animal	Animal	Animal	Animal	Animal
Country		Japan	France	Japan	Japan	Hungary	Germany	France	France	Italy	4 countries ^3^	Switzerland	Japan	Germany	Argentina	Japan	Japan	Japan	Finland	10 Countries ^4^	7 Countries ^5^	Korea	Argentina
Sample No.		70	82	40	310	281	27	31	82	22	262	26	12	16	22	146	37	75	33	183	515	27	61
Reference		[68]	[69]	[76]	[81]	[93]	[92]	[85]	[85]	[79]	[86]	[87]	[88]	[92]	[95]	[96]	[96]	[96]	[54]	[69]	[97]	[90]	[95]
***h*^2^**	**MIRU1**	−	−	−	−	0.250	−	−	−	−	−	−	−	−	−	−	−	−	−	−	0.252	−	−
	**MIRU2**	−	−	−	−	0.224	0.43	−	−	−	−	−	−	0.52	−	−	−	−	−	−	0.564	−	−
	**MIRU3**	−	−	−	−	0.710	0.67	−	−	−	−	−	−	0.7	−	−	−	−	−	−	0.064	−	−
	**MIRU4**	−	−	−	−	0.285	0.07	−	−	−	−	−	−	0	−	−	−	−	−	−	0	−	−
	**MIRU5**	−	−	−	−	−	0.07	−	−	−	−	−	−	0.42	−	−	−	−	−	−	−	−	−
	**MIRU6**	−	−	−	−	−	0.58	−	−	−	−	−	−	0.57	−	−	−	−	−	−	−	−	−
	**MIRU7**	−	−	−	−	−	0.22	−	−	−	−	−	−	0.5	−	−	−	−	−	−	−	−	−
	**TR 25**	0.512	0.33	−	−	0.514	0.36	0	0.26	0.46	−	0.45	0.53	0.5	0.5844	0.517	0.47	0.593	0.55	0.07	0.094	0.446	0
	**TR 32**	0.359	0.3	−	−	0.471	−	0.03	0.17	0.21	−	0	0.21	−	0	0.484	0.497	0.101	0.19	0.59	0.032	0	0
	**TR 47**	0.069	0.35	−	−	−	0.51	0	0.20	0.20	−	0.23	0	0.32	0.3247	0.116	0	0.444	0.43	0.05	−	0	0
	**TR 10**	0.459	0.15	−	−	−	−	0.21	0.13	0	−	−	0.50	−	0.2554	−	−	−	0	0.18	−	0.036	0.0645
	**TR 259**	−	−	−	−	0.589	−	−	−	−	−	−	−	−	−	−	−	−	−	−	0.063	−	−
	**TR 3**	−	0	−	−	−	0	0	0	0	−	0	−	0	0.0909	−	−	−	0	0.005	−	0	0
	**TR 7**	0	0.04	−	−	−	0	0	0	0	−	0	0	0	0.4848	0	0	0	0	0.19	−	0	0.6980
	**TR 8**	−	−	−	−	−	0.49	−	−	−	−	−	−	0.65	−	−	−	−	−	−	−	−	−
	**TR 1685**	−	−	−	−	−	0.51	−	−	−	−	−	−	0.7	−	−	−	−	−	−	−	−	−
	**RD 130**	−	−	−	−	−	0.21	−	−	−	−	−	−	0.12	−	−	−	−	−	−	−	−	−
	**MATR−1**	0.514	−	0.48	0.61	−	−	−	−	−	0.44	0.17	0	−	−	0.494	0.307	0.231	−	−	−	−	−
	**MATR−2** **(= TR 292)**	0.581	0.27	0.59	0.63	−	0	0	0.19	0.46	0.71	0.64	0.53	0.6	0.5714	0.594	0.581	0.577	0.36	0.51	−	0.517	0.5050
	**MATR−3** **(= TR X3)**	0.571	0.72	0.60	0.57	−	0.52	0.64	0.68	0.54	0.67	0.71	0.21	0.56	0.5844	0.519	0.485	0.694	0.66	0.04	−	0	0
	**MATR−4**	0.096	−	0.60	0.47	−	−	−	−	−	0.19	0.23	0.08	−	−	0.08	0	0.593	−	−	−	−	−
	**MATR−5**	0.096	−	0.12	0.20	−	−	−	−	−	0.37	0.52	0.08	−	−	0.079	0	0.615	−	−	−	−	−
	**MATR−6**	0.420	−	0.12	0.55	−	−	−	−	−	0.53	0.41	0.08	−	−	0.492	0.272	0.47	−	−	−	−	−
	**MATR−7**	0.718	−	0.65	0.68	−	−	−	−	−	0.79	0.55	0.23	−	−	0.662	0.498	0.581	−	−	−	−	−
	**MATR−8**	0.376	−	0.49	0.49	−	−	−	−	−	0.51	0.46	0.08	−	−	0.463	0.497	0.657	−	−	−	−	−
	**MATR−9**	0.459	−	0.49	0.65	0.445	−	−	−	−	−	0.66	−	−	−	0.512	0.272	0.494	−	−	−	−	−
	**MATR−10**	−	−	0.56	−	−	−	−	−	−	−	−	−	−	−	−	−	−	−	−	−	−	−
	**MATR−11**	0.431	−	0.58	0.53	−	−	−	−	−	0.63	0.49	0.08	−	−	0.515	0.482	0.576	−	−	−	−	−
	**MATR−12**	0.000	−	0.02	0.09	−	−	−	−	−	0.16	0.47	0	−	−	0.014	0.053	0.026	−	−	−	−	−
	**MATR−13**	0.525	−	0.46	0.53	−	−	−	−	−	0.49	0	0.50	−	−	0.506	0.456	0	−	−	−	−	−
	**MATR−14**	0.480	−	0.53	0.52	−	−	−	−	−	0.50	0.31	0.08	−	−	0.485	0.52	0.409	−	−	−	−	−
	**MATR−15**	0.070	−	0.16	0.01	−	−	−	−	−	0.30	0.17	0.08	−	−	0.118	0	0.517	−	−	−	−	−
	**MATR−16**	0.400	−	0.53	0.50	−	−	−	−	−	0.48	0.35	0.45	−	−	0.508	0.549	0.655	−	−	−	−	−

^1^ When allelic diversity (h) was presented in the references, this information was directly described in this table. Although *h* was not directly presented in the references, when the VNTR type results could be extracted from the results or supplementary data, we calculated their *h* values using these results. ^2^ Allelic diversity index (*h*) = (1 − ∑xi^2^) [*n*/(*n* − 1)], (0 ≤ *h* ≤ 1). In this study, Selander’s formula was adopted and used [75]. Exceptionally, *h* values directly described in Iwamoto et al. (2012) [96] and Radomski et al. (2010) [85] are described by hand. They used one of nei’s formulas, Iwamoto et al. (2012) for Keim et al. (2000) [98], and Radomski et al. (2010) for Nei et al. (1976) [99]. ^3^ Japan, Korea, Netherlands, and USA. ^4^ Argentina, Czech Republic, France, Italy, Netherlands, Slovenia, Sweden, United Kingdom, USA, and Venezuela. ^5^ Denmark, France, Germany, Hungary, Italy, Netherlands, and Slovakia.

## 5. Rep−PCR Procedures and Their Implications in Epidemiological Studies

Rep−PCR analyzes the differences in band lengths by repeatedly amplifying between sequences and scattering them within a chromosome using PCR [100]. There are several evolutionarily conserved repetitive sequences in various strains, including BOX [101], enterobacterial intergenic consensus (ERIC) [102], and the repetitive extragenic palindromes (REP) element [103]. BOX elements are found in *Streptococcus pneumoniae,* and the 57−bp sequence of the BoxA−like elements in several subunits is conserved in various strains [101]. REP (38bp) and ERIC (126bp) are palindromic sequences, which were first discovered in *Escherichia coli* and *Salmonella typhimurium* [104]. These elements are also conserved in various strains that are systematically unrelated [104]. Additionally, polytrinucleotide sequences, such as (GTG)_5_ or (GCC)_5_, which are scattered in the genomes of *E. coli* and *S. typhimurium*, belong to the interspersed repetitive DNA sequences [105]. In mycobacteria, repetitive sequences were identified in *M. bovis* and MTB [106]. The epidemiological and clinical implications of the studies that have been applied to genotyping for MAC using rep−PCR are summarized in Table 3.

### 5.1. Application of Rep−PCR to Epidemiological Investigations of MAC

IS*900*, a MAP−specific IS element with similar sequences in other mycobacteria, limits the detection of MAP by PCR. The development of alternative PCR assays was thus necessary to perform a rapid analysis; as a result, ERIC/IS*900* PCR was developed using both ERIC and IS*900* sequences [107]. ERIC/IS*900* PCR analysis revealed that MAP exhibits a species−specific band pattern, which can be used to distinguish MAP from other mycobacteria [107]. However, all the MAP strains that were previously classified using RFLP analysis could not be identified by ERIC/IS*900* PCR analysis and cannot be used as an alternative to RFLP analysis [107]. However, this study suggested that a repetitive sequence for MAC can be used for epidemiological analysis. The mycobacterium strain genotyping kit from DiversiLab was released in 2003 for rep−PCR of the BOX, ERIC, and REP sequences, as reported by Versalovic et al. (1991) and Koeuth et al. (1995) [108]. This kit was used for a comparative analysis of IS*1245*−RFLP and rep−PCR patterns of *M. avium*. Additionally, the rep−PCR analysis was used independently for *M. intracellulare*. There was 96% similarity between RFLP and rep−PCR patterns, except for the strains with little or no IS elements [108]. It was estimated that the ability of rep−PCR was similar to or higher than that of RFLP, and that the complex pattern of *M. intracellulare* could be identified as a result [108].

Additionally, rep−PCR was performed directly using the primers designed by Versalovic et al. (1991) and Koeuth et al. (1995) instead of the commercial kit, classified 176 *M. intracellulare* isolates into only three types compared to the nine types classified by VNTR, indicating that the primers designed by Versalovic et al. (1991) and Koeuth et al. (1995) may not be suitable for the diagnosis of a *M. intracellulare* relapse [63]. Meanwhile, the study by Otsuka et al. (2004) found that trinucleotide repetitive sequence−based PCR (TRS−PCR) could be used to identify not only *M. tuberculosis* and *M. bovis* strains, but also *M. avium* strains [109]. This led to the studies identifying strains in MAC or the same species strains of MAC via (CCG)_4_−PCR, revealing a high diversity index of 0.979 and reproducibility of 95.1%. These findings indicated that the combined use of MIRU−VNTR and (CCG)_4_−PCR analysis could be used for the identification of *M. avium* [89].

### 5.2. Implication of Rep−PCR Methods in MAC Epidemiological Investigations

In subsequent experiments, *M. avium* clinical isolates (not MAP) were collected from various countries to investigate the current distribution of the MAH 104 strain, which was first isolated from California in 1983 [110]. Large sequence polymorphism PCR (LSP PCR) was performed to confirm the presence of four hypervariable genomic regions. The rep−PCR analysis of 19 strains with the same LSP types as MAV104 revealed that 10 strains had the same pattern as MAH 104 [110]. As these 10 strains were collected from 10 different patients over 17 years at five clinical sites in the West Coast of the United States, it was confirmed that MAV104 still results in disease in many patients in the West and that its genotype remains consistent over time [110].

Subsequently, a study was conducted to determine whether *M. avium* detected in patients via bronchoscopy were, in fact, pathogens or contamination from the bronchoscope [111]. Of the 22 *M. avium* clinical isolates and 56 *M. intracellulare* clinical isolates, 5 (23%) and 42 (75%) isolates were similar to those detected in the bronchoscope and the water in the preparation room used to wash the bronchoscope, respectively [111]. Notably, over 10% of MAC isolates were able to survive even after 3 to 4 h of exposure to hot water with a temperature of 60 °C or above [112], suggesting that the samples isolated from patients have a high probability of being contaminated by a bronchoscope washed with water, and that even healthy individuals could be exposed to MAC during bronchoscopy [111]. Following this study, the plumbing lines that supplied water to the homes of the patients were examined. Of the 37 households studied, 17 (46%) had the same NTM species as the isolate obtained from the patient, including MAC [61]. Additionally, the rep−PCR analysis revealed that these strains were the same, which indicated the possibility of MAC infection among households [61]. However, one study reported that infection relapse in HIV patients with *M. avium* bacteremia post−high−efficiency AIDS treatment was caused by the same strain [113]. The rep−PCR pattern of *M. avium* isolates exhibited 64–78% consistency with that of the control isolates, while the rep−PCR patterns exhibited 99.5% consistency among the *M. avium* isolates [113]. As these previous studies suggest that MTB can be latent in adipocytes, further studies are necessary to confirm whether MAC is also latent, in order to develop strategies for the prevention of MAC relapse in patients with AIDS [113]. Similarly, rep−PCR analysis revealed that the continued relapse of MAH in patients with hypersensitivity pneumonitis was caused by hot tubs installed in their homes. Upon discontinuation of hot tub use, the health of the patient improved. This demonstrated the importance of rep−PCR analysis in determining patient treatment [114].

### 5.3. Clinical Application of MAC Rep−PCR

Rep−PCR is widely used in clinical studies due to its simplicity. Recently, the difference in treatment outcome or relapse according to the phenotypes of MAC lung disease (MAC−LD) was evaluated in 481 patients with MAC−LD, demonstrating a 29% relapse rate among patients with good treatment outcomes. Additionally, this study demonstrated that the phenotype of nodular bronchiectasis was a significant risk factor for relapse and that 74% of relapses were due to reinfection by other strains [115]. Daily and intermittent treatments with antibiotics for recurrent noncavitary nodular bronchiectatic (NB)−type MAC−LD were compared by rep−PCR analysis, which revealed that reinfection by strains with a new genotype was the cause for 86% of the case of relapse and the antibiotic toxicity of daily treatment. This indicated that intermittent treatment was appropriate for this disease [116]. Subsequently, a study was conducted to determine whether antibiotic resistance or reinfection was responsible for the positive culture exhibited by patients with refractory MAC−LD, even after treatment with macrolide antibiotics for over 12 months. The analysis revealed that 22% of patients treated for an average of 33 months were resistant, and 73% of relapsed patients were re−infected with a new strain of MAC. This confirmed that reinfection was a more important factor in refractory MAC−LD than macrolide resistance [41].

Currently, the rep−PCR analysis of the MAC complex is mainly being performed using commercial kits. Additionally, the primers designed by Versalovic et al. (1991) and Koeuth et al. (1995) are commonly used. However, the commercial product was discontinued and the primers designed by Versalovic et al. (1991) and Koeuth et al. (1995) are considered to have a low identification capability. As rep−PCR analysis could be used to diagnose the cause of relapse more quickly and more easily than PFGE or VNTR methods, it is important to develop primers that can identify MAC complex more effectively than the currently available options.

## 6. Conclusions and Perspectives

MAC is the most frequently isolated species that causes human disease among NTM across the world. With an increasing understanding of the genetic diversity, differential pathogenicity, and varied infectious sources of MAC, new MAC species and subspecies are constantly being identified. Certain environments, such as in water and soil, likely become a niche for *M. avium* and *M. intracellulare*. *M. avium* that is excreted from infected animals contaminates the environment, but no evidence exists for similar environmental contamination by *M. intracellulare*. However, the transmission mode of MAC with specific genetic characteristics is not yet clearly defined, and more reliable and feasible genotyping methods of MAC are urgently needed. As summarized in this review, several genotyping methods based on unique genetic markers of MAC species and subspecies might improve our understanding of estimating the infection pathway among animals, humans, and the environment, and evaluation of the treatment strategies based on the treatment outcomes and the pattern of recurrence of MAC infection. The establishment and application of criteria for the selection of the appropriate genotyping techniques for each MAC strain and the relevant epidemiological investigations will allow for the improvement of public health preventive measures and for an increased effectiveness of MAC patient management.

**Table 3:** List of rep−PCR based genotyping methods applied to *Mycobacterium avium* complex.

Strain	Origin		Country	Sample No.	Primers	Epidemiologic Characteristics	Reference
MAA	Animal	cat, cattle, chukar, deer, dog, hobby, horse, pig, polecat, and peat	Sweden	16	s535 (IS*900* specific outward primer), ERIC2	- As a result of ERIC/IS*900* PCR using the ERIC sequence and IS*900* for the epidemiological analysis of MAP, MAP showed species−specific band patterns, which can be used as a method for discriminating it from other mycobacteria. - However, the MAP strains that were discriminated by RFLP cannot be distinguished and thus cannot be used as an alternative genotyping method of RFLP.	[107]
MAP	Human & animal	bovine, deer, goat, ovine, human	USA Europe	60	s535 (IS*900* specific outward primer), ERIC2		
MAS	Animal	−	Norway	1	s535 (IS*900* specific outward primer), ERIC2		
*M. intracellulare*	ATCC			3	s535 (IS*900* specific outward primer), ERIC2		
MAA	Human & environment	patients and environment	USA	28	DiversiLab Mycobacterium kit	− The genetic analysis pattern of RFLP and rep−PCR is 89% concordant, such that the genetic discrimination of rep−PCR is equal to or better than that of RFLP.	[108]
*M. intracellulare*	Human	patients	USA	8	DiversiLab Mycobacterium kit	− Eight *M. intracellulare* clinical isolates showed different patterns with rep−PCR.	
*M. avium*	Human	Patients	USA Canada Netherlandss Brazil	207	DiversiLab Mycobacterium kit	− The isolates from ten different patients at five clinical sites in the western US were genetically identical to the standard strain MAH 104. − The bacterium is involved in causing disease in many patients in the western US, indicating that the genotype of the pathogen is stable over time.	[110]
*M. avium*	Human & environment	Patients, bronchoscopy preparation laboratory	USA	22 clinical, 16 laboratory	Cangelosi et al., 2004	- Water and biofilm samples collected from the bronchoscopy preparation laboratory yielded mycobacteria, including *M. avium* and *M. intracellulare*. - It is assumed that infection with 5/22 (23%) *M. avium* isolates and 42/56 (75%) *M. intracellulare* isolates is the result of a contaminated water supply	[111]
*M. intracellulare*	Human & environment	Patients, bronchoscopy preparation laboratory	USA	56 clinical, 4 laboratory	Cangelosi et al., 2004		
*M. avium*	Human & environment	NTM patients, household sample	USA Canada	9 clinical, 10 environmental	Cangelosi et al., 2004	− Of the 17 strains of MAC strains isolated during 2007–2009 in water distribution system of 37 patients, seven strains had the same genotype as the patients (matching rate 41%).	[61]
*M. intracellulare*	Human & environment	NTM patients, households sample	USA Canada	6 clinical, 10 environment	Cangelosi et al., 2004		
*M. avium*	Human & environment	Patients with chronic rhinosinusitis, household sample	USA	6 clinical, 33 environment	Cangelosi et al., 2004	− Three isolates from household samples of chronic rhinitis patients previously infected with NTM were genetically associated with the isolate identified in the patient. − This suggests that chronic rhinitis patients may be infected with NTM in their own household.	[43]
*M. avium*	Human	Patients with HIV positive	France	8	DiversiLab Mycobacterium kit	− The genetic reconciliation between 2002 and 2009 isolates was 99.5% in patients who relapsed 7 years after their first infection by *M. avium*.	[113]
*M. avium*	Human	clinical isolates	Poland	33	N_6_(CCG)_4_	− The discrimination index of (CCG)_4_−PCR for *M. avium* was 0.979, which was higher than 0.945 obtained for MIRU−VNTR (TR32, TR292, TRX3, TR25, TR7, TR10, and TR47). − However, MIRU−VNTR was able to distinguish certain strains that (CCG)_4_−PCR could not. As such, a combination of (CCG)_4_−PCR and MIRU−VNTR is proposed for *M. avium* genotyping.	[89]
MAH	Human & environment	Patients, household sample	Netherlands	5	DiversiLab Mycobacterium kit	− MAH is still detected after treatment of hypersensitivity pneumonia, suggesting relapse in infection due to the use of contaminated hot tubs found in the patients’ houses. − Rep−PCR analysis confirmed that the strain isolated from the patient was the same as the hot tub isolate. Thus, after stopping the use of the hot tub, no further MAH relapses took place.	[114]
*M. intracellulare*	Human	Patients with nodular bronchiectasis	USA	176	Versalovic et al. 1991	− Genotyping was performed using PFGE, MIRU−VNTR (MIRU 3, MIN 18, 19, 20, 22, 31, 33), rep−PCR, and ITS region sequencing using 176 *M. intracellulare*. − Thecombination of VNTR and 16S multiplex PCR has a similar reliability to PFGE. − Rep−PCR is excluded because of its low level of discrimination power of rep−PCR in the identification of relapse.	[63]
*M. avium*	Human & environment	patients and environment	Brazil USA Canada, Netherlands	127 clinical, 52 environment	Bacterial Barcodes mycobacterial kit (Athens, GA)	− As a result of comparing the discrimination between several types of genotyping methods(hsp65 sequencing, LSP, 4−locus MIRU, and 8−locus MIRU) including rep−PCR, LSP−MVR (a combination of LSP and MIRU−VNTR)was selected as the high resolution genotyping method. − The HGDI value of rep−PCR was 0.97.	[117]
*M. avium*	Human	Patients with NB, cavitary NB, fibrocavitary disease	South Korea	31	DiversiLab Mycobacterium kit	- In 481 patients with MAC lung disease who received antibiotic therapy for over 12 months were associated with re−infection by other bacteria (74%), while 26% of recurrence resulted from infection by the same bacteria (26%), according to the rep−PCR results. - The NB form was also determined as a significant risk factor for the recurrence of NTM lung disease.	[115]
*M. intracellulare*	Human	Patients with NB, cavitary NB, fibrocavitary disease	South Korea	34	DiversiLab Mycobacterium kit		
*M. avium*	Human	Patients with MAC lung disease	South Korea	52	DiversiLab Mycobacterium kit	- The therapeutic effectiveness of intermittent antibiotic therapy was evaluated in patients previously treated for MAC lung disease and receiving antibiotic treatment for recurrent noncavitary NB MAC lung disease -86% (12/14) of relapsed patients were found to be infected with a new strain of MAC -As such, intermittent antibiotic therapy was suggested intermittent antibiotic therapy as to be a reasonable treatment strategy for recurrent noncavitary NB MAC lung disease.	[116]
*M. intracellulare*	Human	Patients with MAC lung disease	South Korea	46	DiversiLab Mycobacterium kit		
*M. avium*	Human	Patients with Refractory MAC lung disease	South Korea	80	DiversiLab Mycobacterium kit	- In 72 patients with refractory *M. avium* complex lung disease (MAC−LD) who received antibiotic therapy, including macrolides, for over 12 months, macrolide resistance was found in 16 patients (22%). - Of the 49 patients recorded before and after treatment, 24/49 (49%) patients were found to be infected with a new MAC strain, while 12/49 (24%) patients were infected by both the original and new strains. Only 13/49 patients (27%) showed persistent infection by the original MAC strain. - In conclusion, refractory MAC−LD is generally caused by reinfection of other strains rather than the relapse of the original strain, which is thought to be due to intermittent macrolide resistance.	[41]
*M. intracellulare*	Human	Patients with Refractory MAC lung disease	South Korea	120	DiversiLab Mycobacterium kit		
